# Physicochemical Changes in Bone Bioapatite During the Late Postmortem Interval Pre- and Post-Burning

**DOI:** 10.1177/00037028221085600

**Published:** 2022-06-17

**Authors:** Emese I. Végh, Nicholas Márquez-Grant, Rick J. Schulting

**Affiliations:** 1 Research Laboratory for Archaeology and the History of Art, 6396 University of Oxford, Oxford, Oxfordshire, UK; 2 Defence Academy of the United Kingdom, Cranfield Forensic Institute, 547683 Cranfield University, Cranfield, Bedford, UK

**Keywords:** Cremation, electron microprobe, Fourier transform infrared, FT-IR, taphonomy, potassium

## Abstract

Postmortem chemical transformation of bone bioapatite can take place during early diagenesis, resulting in a more thermodynamically stable mineral phase. This paper examines the impact of a one year postmortem interval on unburnt and burnt bone’s structural and chemical alterations. This question is of importance for the reconstruction of funerary practices involving cremation in the archaeological record, as well as forensic anthropological investigations. Fleshed pig (*Sus scrofa*) tibiae were left exposed in a field, then collected at 14, 34, 91, 180, and 365 day intervals prior to being burnt in an outdoor fire (≤750 °C bone temperature). Fresh (fleshed) tibiae acted as unburnt and burnt controls. Also included in the study were two cremated human bone fragments from Middle–Late Neolithic (ca. 3300–2500 BCE) Ireland. Samples were analyzed for major and trace elements using an electron microprobe wavelength dispersive analyzer and molecular structures using Fourier transform infrared spectroscopy. Linear regression, principal component analysis, linear discriminant analysis, and multivariate analysis of variance were performed for statistical analysis. Results indicate that the concentrations of elements associated with extracellular fluid (K, Na, and Cl) change with the postmortem interval (PMI) and survive burning. K values under 0.07 ± 0.01 wt% in the inner and mid-cortical zones of burnt bones suggest that bones were not burnt immediately after death. Using this criterion, results from the archaeological samples would indicate a PMI of at least weeks to months prior to cremation. Ca, P, Fe, Al, Si, and Sr are not significantly altered with burning, and Fe, Al, Si, and Sr are also unaffected by the PMI. In unburnt bones increased crystallinity and carbonate loss are detectable in <1 year, but both are obscured by burning. Structurally, the carbonate to phosphate ratio (C/P), the phosphate high temperature, and cyanamide to phosphate (CN/P) are the most useful ratios for discriminating between unburnt and burnt bones.

## Graphical abstract

**Graphical Abstract:**
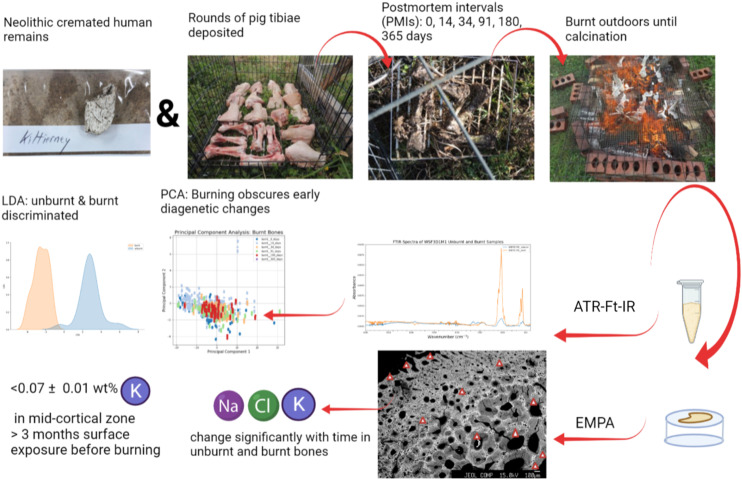

## Introduction

Thermally altered human bones occur in both forensic and archaeological contexts. Burnt bones can result from accidental fires or arson, or to mask the victim’s identity as well as the cause and manner of death in a homicide case.^1^ Since antiquity, cremation has been a common funerary practice in many cultures, though there are often questions regarding the state of the corpse at the time of burning. In several tombs and burials of Neolithic, Bronze, and Iron Age Ireland,^2–5^ Scotland,^6,7^ England,^8–11^ and France,^12^ for example, human remains may have been excarnated before cremation, while in other cases bodies seem to have been burned soon after death.^3–5^ Some of this interpretation is based on heat-induced fracture patterns including thumbnail fractures and warping, associated with the burning of fresh bones in early studies.^13–18^ However, more recent research has shown that these fractures do not depend on the presence of soft tissues, but rather on collagen content and distribution.^19,20^ Recent attempts to identify bone decomposition prior to burning through histotaphonomical analysis^21^ may be unreliable.^22^ Identifying when burning took place after death presents a challenge, one addressed here through a study of the physicochemical changes of bones pre- and post-burning up to one year post-deposition.

If changes in elemental concentrations to bone are detectable after a short period of deposition/postmortem interval (PMI; in the current study two weeks to one year) and they survive burning, it can be hypothesized that it is possible to inform on the pre-burning condition, that is, on whether bodies were fresh or partly decomposed when burnt. Although effects of diagenesis on the elemental concentration had been investigated,^23–25^ no studies have examined these changes after exposure to heat. Therefore, this study aims to assess the potential of compositional and structural changes in unburnt and burnt bone to help distinguish between fresh and variably taphonomically altered bones within a defined post-mortem interval. The results of this experiment are compared to the chemical composition of archaeological (Neolithic) cremated bones. Our hypothesis based on previous studies (e.g., Keenan and Engel,^24^ Walden et al.^25^) is that these changes can occur weeks following deposition by the loss of endogenous elements in vivo and from the uptake of exogenous elements from the environment. We further hypothesize that these changes will be observed in the burnt bones since inorganic mass loss is negligible in bones burnt at up to 900 °C.^26^

### Taphonomic Alterations to the Elemental Concentration and Structure of Bone

Bone is a composite material consisting of ∼55–70 wt% mineral, ∼20–35 wt% organic carbon, and ∼10–15% water.^27–29^ Hydroxyapatite [HAp, Ca_10_(PO_4_)_6_(OH)_2_] is the basis of all biologically formed apatite, while bioapatite [(Ca,Mg,Na)_10–x_[(PO_4_)_6–x_(CO_3_)_x_](OH)_2–x_] is a carbonate-containing variety of HAp, which forms the inorganic component of bone.^28,30,31^ Bioapatite is chemically more complex than HAp; it is non-stoichiometric, and can incorporate a wide variety of elements into its structure.^28,31–33^

The chemical profile of bone (Table S1, Supplemental Material) results from the combination of the pre-mortem (e.g., diet and inhalation) and postmortem (e.g., degradation and soil) incorporation and release of elements. However, it is modified by taphonomic (post-mortem) factors, which can be natural (e.g., weathering, mineral replacement, and microbial attack) or anthropogenically induced (e.g., inhumation, sub-aerial exposure, disarticulation and defleshing, and burning).^34^ Early decomposition includes the hydrolysis of collagen, and the first biological, chemical, and structural changes to bioapatite.^35,36^ Bioerosion causes increased surface areas which either encourages mineral dissolution or enhances the movement of ions and substitutions, eventually leading to a more thermodynamically stable mineral phase.^37^ Organic loss has been associated with increased ion transport^36,37^ due to the open pore spaces between the mineral phase, facilitating the movement of fluids, microorganisms, and dissolved ions,^35^ unless pore spaces are blocked by ingress of trace elements from the environment.^38^ Fossils, the resulting mineral phase from late diagenesis, have a wide variety of substitutions, but most commonly have F- and Ca-enriched apatite phases^35,39,40^ or CO_3_^2–^ loss.^41^ An experiment in which modern bones were exposed for up to 30 years on soil surfaces in Kenya showed that crystal sizes increase in number and size with time.^42^

Bone composition is susceptible to changes as soon as bone is exposed to the environment, which has been documented in unburnt modern,^24,25^ archaeological,^43^ and fossil bone,^37^ but regarded as negligible.^44^ Bioapatite can accommodate various substitutions at every site of the crystal lattice, which makes it one of the most diverse minerals.^24,45^ Cations (metals) substitute for Ca(I) and Ca(II),^46^ while anions for phosphates, carbonates, and hydroxyls. Trace elemental uptake due to ion exchange can occur after one week, while after a year a more thermodynamically stable mineralogical phase shift to F- and Fe-enriched apatite phases can be seen.^24^ Non-uniform, nonlinear increases (Mn, Fe, Ca, and F) and decreases (Cl and Na) were also seen in bone buried >1 year. However, the calcium to phosphate ratio (Ca/P) remains constant during putrefaction,^47,48^ while a decrease of carbonate to phosphate (C/P) has been identified after burial for one year. The latter is due to either carbonate loss (A and B), increased phosphate content, or the loss of organic material in bone.^24^

Much of the work on trace element uptake into bone has revolved around rare earth elements and uranium,^49–51^ which are taken up by bioapatite over 10^3^–10^6^ years post-mortem.^50^ In modern bone, Ca, P (from bioapatite), Fe, Na, K (associated with bodily fluids), F, Mg, Al, Si, Cl, and Mn (associated with trace elements from soil) are usually studied. Fe, Al, and Mn are often abundant in weathered soils but are less likely to enter into solution.^52^ If this does occur, Al and Mn do not only affect the outermost layers of bone, but are measurable as contaminants in the mid-cortical region in archaeological bone.^53^ Fe is stored in vivo in blood, bodily organs,^25,54^ with a minuscule amount being surface-bound in bone.^47,54^ The decrease of Na and K has been linked to the dehydration of bone during decomposition.^25^ Mg is surface-bound in bioapatite, and although it has been shown not to be much affected by diagenesis, very small amounts can be incorporated into the bioapatite lattice.^25^ In buried bone cations diffuse inward toward the pores from the outer cortex.^51^ Adsorption will cease once equilibrium is reached with the surrounding pore waters,^55^ also evidenced by the slowing and halting of trace element leaching during fossilization.^56^ The rate of approaching equilibrium with pore waters is dependent on the element concentration, mobility in the environment, and the adsorption coefficient (i.e., a constant relating to the speed a molecule will bind to the surface of bioapatite at defined conditions).^51,55^

### Heat Changes to the Physicochemical Structure of Bone

Generally, heat-induced changes in bone go through the following stages: (i) dehydration of the bone with the breakage of hydroxyl bonds and water removal; (ii) organic component (lipids and proteins) decomposition around 400–500 °C; (iii) inversion, marked by carbonate loss (CO_3_^2–^ disappears completely at ∼ 700 °C); and (iv) fusion of the crystals (>700 °C) when the crystallites grow in size and OH^–^ and PO_4_^2–^ rearranges within the pores which are now vacant of organics and water.^19,57^

Many studies have investigated the elemental compositional changes of burnt bone in archaeological and experimental contexts,^32,43,58–61^ and for biomaterials research (e.g., Holden et al.,^62^ Rogers and Daniels^63^). During the burning process, bone is susceptible to chemical substitutions.^64^ Although in theory, most inorganic elements in bone should not change at temperatures possible to reach in an outdoor fire, the inorganic mass loss has been shown to fluctuate around 2.8 wt% independent from temperature during heating up to 900 °C.^26^ Various heat-induced elemental changes have been documented (Table S2, Supplemental Material).

The main structural changes observed include the reordering of phosphate, the decrease/loss of carbonate, and increased crystallinity,^26,60,65^ which start to occur at 600/700 °C.^26,66,67^ Extensions to the α-lattice and changes to the c-lattice parameters are accompanied by the release of lattice carbonate and possibly water, which results in an increase in crystal size and crystallinity.^68^ At temperatures around 730 °C, a new phase of beta-tricalcium-phosphate (β-TCP) appears in bioapatite, which remains stable at high temperatures.^69^ The appearance of β-TCP signals the thermal decomposition of the apatite phase, which happens due to non-stoichiometric substitutions/P ratio (i.e., <1.67).^69,70^ Another secondary phase by-product is CaO, which appears at >700 °C ^71^ is due to the thermal decomposition of CaCO_3_.^72^

As regards diagenetic processes, most research has investigated chemical changes to bone post-burning and after deposition,^43,58^ which was thought to be similar in cremated bone as in unburnt bone in earlier research.^49,58^ However, these studies were observational with no controls,^49^ and only referring to calcium.^58^ Bioapatite is less reactive post-deposition in an archaeological timescale^42,73^ and especially after burning because the crystals transform into a more stable thermodynamic phase;^74^ thus, the inorganic structure seems to remain stable post-burning. The amount of organic material in bone prior to burning appears to affect thermal decomposition as traced through OH^–^, carbonate (type A and B), β-TCP, tetracalcium phosphate (TetCP), loss of CO_2_ and water content,^75^ and presence of cyanamide.^74^

## Materials and Methods

The material and experimental protocol employed here follows that of a parallel study,^22^ which examined the histotaphonomic features in unburnt and burnt bones to inform on the PMI. Fleshed pig (*Sus scrofa domesticus)* tibiae (*N* = 22) were allowed to decay exposed within a cage for 14 (*N* = 5), 34 (*N* = 5), 91 (*N* = 5), 180 (*N* = 5), and 365 (*N* = 2) days prior to burning on an outdoor fire in Wytham Woods, Oxfordshire, UK. Fresh pig tibiae (*N* = 10) served as controls throughout the study, without being exposed. Over time the exposed bones became partially submerged in soil and vegetation; however, experimental studies have found no significant differences in elemental concentrations in buried and surface-exposed bones.^25^

Wytham Woods is located in a temperate climate with annual precipitation averaging 717 mm and mean winter and summer temperatures of 1.6 °C and 20.3 °C, respectively. Soil pH ranges widely from 3 to 7.^76^ Bones were burnt on an outdoor fire, which was executed in the same manner with each event lasting from 2.5–3 h until the bones were deemed to be fully calcined based on their appearance. A thermocouple was used to directly monitor bone temperature, with a mean of 553 °C and a recorded maximum of 751 °C.^22^

Bone samples were taken from diaphyses before and after burning to maintain consistency with other studies of elemental concentration.^25^ The unburnt samples were defatted in a 2:1 chloroform:methanol mixture. The composition and atomic-level structure of the bones were analyzed from: (i) polished resin blocks using wavelength-dispersive X-ray spectroscopy (WDS) on an electron microprobe (EMP) and (ii) powders using Fourier transform infrared spectroscopy (FT-IR) in attenuated total reflection (ATR) mode. Electron microprobe wavelength dispersive allows spatially resolved analysis of a single bone. This approach is essential since the outer layer of the bone may have a different distribution of elements than the mid- or inner cortical bone adjacent to the medullary cavity (cf. Gallello et al.^43^). FT-IR has been used previously to determine bone composition (organic and inorganic) and bone structure, such as the infrared splitting factor (IRSF), which provides insights into bone crystallinity (e.g., Keenan and Engel,^24^ Snoeck et al.^74^).

Additionally, two cremated human bone fragments from burials 1 and 7 at a Middle/Late Neolithic (ca. 3300–2500 cal BCE) stone circle in Kiltierney, Co. Fermanagh, Northern Ireland,^77^ were included. The fragments presented thumbnail fractures, suggestive of high bone collagen content at the time of burning.^20^ They were analyzed with EMP, to compare their bone chemistry to the experimental results.

### Fourier Transform Infrared Spectroscopy

Fourier transform IR was used to retrieve structural and molecular information from the unburnt and burnt bones. This has been frequently used to investigate both the diagenetic pathways in osseous material,^78–80^ to differentiate between heated bone at low, medium, and high temperatures,^81,82^ and to characterize modern and archaeological burnt bone.^74,83^ ATR was chosen instead of KBr discs, as it has been shown to provide more reliable measurements and fewer chemical modifications.^79,81,84,85^

The powdered pre- and post-burnt samples were measured with a Cary 600 series FT-IR spectrometer 5.3 (Agilent Technologies, USA). A diamond crystal accessory was utilized with an operational range of 30 000 to 200 cm^–1^. Resolution Pro FT-IR software (Agilent Technologies, Santa Clara, California, USA) was used for automatic software correction and for generating the backgrounds and the raw data for each sample. After the spectrometer was aligned, a resolution of 4 cm^–1^ was chosen. Sixty-four scans were taken of each sample with scan range set between 4000 and 400 cm^–1^, which represents mid-IR spectrum analysis. The background was measured after at least every second sample. Before the sample was placed on the accessory, the live spectrum was monitored to ensure no background noise. Baseline corrections were made at around 450, 800, 900, and 1200 cm^–1^. The calculated ratios included those from the literature: the crystallinity index/splitting factor (IRSF),^20,60,66,86^ amide to phosphate (APR),^87^ carbonate (type B) to phosphate (C/P), amount of type B carbonate (BPI),^81^ type A carbonate to phosphate (API),^81,88^ type B to type A carbonates (C/C), cyanamide to phosphate (CN/P),^74^ carbonyl to carbonate (CO/CO_3_), amide I to phosphate (N/P) ratios, and the phosphate high temperature (PHT)^67^ (Table S3, Supplemental Material). Additionally, the presence of β-TCP was noted when present as a peak at 1123 cm^–1^ and as a shoulder at 547 cm^–1^.^89^ Presence of β-TCP implies that burnt bone reached >700 °C. If this β-TCP peak is present at 1123 cm^–1^, IRSF loses its usefulness in estimating crystallinity. It was also suggested that the presence of this peak might be associated with Mg substitution for Ca in the bone.^89^ Type A carbonates (∼1540 cm^–1^) were not evaluated in unburnt bone because its peak overlaps with amide II.^88^ However, it was included in the burnt bone spectra evaluations because organics were removed by heating. The peak heights were evaluated by observing the maximum height from the data at around the associated absorbance using Jupyter notebook in Python programming language. If a slight frequency shift was present (±5 cm^–1^), the maximum height between this interval was recorded for the ratio calculations and the extent of shift noted.

### Electron Microprobe Analysis

A total of 58 unburnt and burnt bone fragments were embedded undecalcified into epoxy cold mounting resin and a catalyst (Spectrographic Ltd., UK) and placed under vacuum in a desiccator to ensure diffusion of the resin into bone pores. Mounted blocks were then ground with carbon papers of progressively finer grit size (800, 1200, and 2500) using a grinding and lapping machine (Buehler, USA). The blocks were then polished on a Buehler wheel using 3 mm diamond paste (DP-Paste M, Struers A/S) and a satin woven acetate polishing cloth (DP-Dac, Struers A/S) for 30 min following another 5 min cycle using another polishing cloth with 1 mm monocrystalline diamond suspension (MetaDi, Buehler). The mounts were cleaned in an ultrasonic water bath after each phase, then finished with another round of ultrasonic bath in petroleum ether (40–60 °C, analytical reagent grade, Fisher Scientific). Samples were carbon-coated using a carbon evaporation coater (HHV Auto 360), the carbon acting as a conductive layer to prevent charging.

Major and trace element compositions were determined using a wavelength-dispersive Jeol JXA-8200 electron microprobe at the Research Laboratory for Archaeology and the History of Art, University of Oxford, UK. An accelerating voltage of 15 kV, 10 nA beam current, and 10 μm beam were used to analyze the polished samples. Peak count times were 30 s for P, Al, Na, Si, K, and Ca; 40 s for Cl; and 60 s for Mg, Sr, Fe, and Mn. Background counts were collected for half the peak count time either side of the peak.^90^ The microprobe was calibrated using a range of mineral standards, with accuracy verified by analyzing apatite secondary standards (Durango, Bamble, and Wilberforce)^91^ and previously analyzed secondary bone standards provided by the Directorate Earth and History of Life, Royal Belgian Institute of Natural Sciences, at the start of every run. Reference materials were within ±1 standard deviation of the preferred values.^92^ Analytical errors of the electron microprobe analysis (EMPA) and detection limits for the measured elements are provided in Table I. K and Mg were in much higher concentration in the samples than in the secondary standards, meaning that their error rates presented a lower value. Thus, the standard deviation percentage (SD%) was measured for these elements which were 37.87 SD% for K and 3.83 SD% for Mg. A total of 617 measurements were taken from the outer cortical layer (OC), mid-cortical region (MC), inner cortical layer (IC), and around vascular canals (HC) on the transverse bone sections. This excludes 82 points that were removed from the dataset because the sum of elements was less than expected in bone (total elements measured <50%) due to errors in locating and focusing the electron beam.

**Table I.** The coefficient of variation and detection limits of the measured major and trace elements in the secondary standards.

Element	Secondary standards	Confidence interval (CV)	Detection limit
P	Bamble, Durango, Bone	0.79	0.07
Na	Bamble, Durango, Bone	6.90	0.02
Cl	Bamble, Durango, Bone	3.49	0.01
K	Bone	17.22 (SD%: 37.87)	0.01
Fe	Bamble, Durango, Bone	33.99	0.01
Al	Wilberforce	1.71	0.03
Si	Bamble, Durango, Wilberforce	16.81	0.04
Sr	Bamble, Durango, Wilberforce, Bone	57.62	0.03
Mn	Bamble, Durango, Wilberforce, Bone	65.20	0.01
Mg	Bamble, Wilberforce, Bone	2.82 (SD%: 3.83%)	0.02
Ca	Bamble, Durango, Bone	0.51	0.01

### Statistical Analysis

The absorption peaks, ratios from the FT-IR, and the elemental concentration EMPA data were analyzed using Python programming language. Descriptive statistics and linear regression were used to measure the strength of the relationship between the measured changes and the PMI in both the unburnt and burnt bones, with statistical significance assessed by associated *p*-values (α = 0.05). The null hypothesis was that parameter X does not increase with PMI. The coefficient of variation (CV) was used to evaluate the scattering of parameters in each group with different PMI and burning status.

Principal component analysis (PCA) and linear discriminant analysis (LDA) were used to identify the main elements of interest and FT-IR ratios were used to differentiate between PMI groups and burning states. LDA was a better statistical model for the purposes of this study. A priori categories in this study were state (unburnt versus burnt), PMI (0–365 days), and zone (outer-, mid-, inner-cortical, and around vascular canals). The confidence score of each sample determined the parameter based on which they were placed into one of these categories. Multivariate analysis of variance (MANOVA) was used to examine for statistical differences on one dependent variable (PMI) by independent grouping variables (EMPA and FT-IR datasets) in Statsmodels Python library. This was coupled with pairwise post hoc multivariate comparison testing (MCT) to identify which specific groups significantly differed from one another.

Datapoints below detection points (Table IV) were entered as 0.00 in the EMPA data. Both FT-IR and EMPA dataframes were scaled for the analyses using Scikit-Learn Python library. All data analyses were performed in Jupyter Notebook in Python programming language.

**Table IV.** Linear regression and multivariate comparison test on the electron microprobe analysis data. Correlation Coefficient (*r*) and *p*-value (*p*) are given for each analyzed element in unburnt and burnt bone for the linear regression. H_0_ = X parameter does not increase with postmortem time period.

Element	Location	Linear regression				Multivariate comparison test (MCT)		
		Unburnt		Burnt		PMI		State
		*r^2^*	*p*	*r^2^*	*p*	Unburnt	Burnt	
P_2_O_5_	OC	0.602	0.206	0.204	0.699	0.419	**0.000	**0.001
	MC	0.246	0.638	–0.276	0.597			
	IC	0.217	0.68	–0.572	0.236			
	HC	0.063	0.905	–0.428	0.397			
	All	0.277	0.19	–0.284	0.179			
Na_2_O	OC	–0.172	0.745	–0.676	0.141	0.061	**0.000	**0.001
	MC	–0.436	0.388	–0.873	**0.023			
	IC	0.561	0.247	–0.889	**0.018			
	HC	0.236	0.652	–0.872	**0.023			
	All	–0.051	0.813	–0.744	**0.000			
Cl	OC	–0.397	0.436	0.830	**0.041	**0.000	**0.000	**0.001
	MC	–0.099	0.852	0.702	0.120			
	IC	–0.142	0.789	0.843	**0.035			
	HC	–0.009	0.986	0.801	0.055			
	All	–0.162	0.449	0.690	**0.000			
K_2_O	OC	–0.655	0.158	–0.54	0.269	**6.05E-26	**9.15E-23	**0.001
	MC	–0.603	0.205	–0.666	0.149			
	IC	–0.834	**0.039	–0.810	**0.050			
	HC	–0.551	0.257	–0.804	0.054			
	All	–0.552	**0.005	–0.578	**0.003			
FeO	OC	–0.413	0.416	–0.397	0.436	0.767	0.302	0.839
	MC	0.614	0.195	–0.097	0.855			
	IC	–0.153	0.773	0.011	0.983			
	HC	–0.493	0.321	–0.395	0.438			
	All	–0.160	0.454	–0.251	0.238			
Al_2_O_3_	OC	–0.304	0.558	–0.027	0.96	0.633	0.209	0.900
	MC	–0.497	0.316	0.502	0.311			
	IC	–0.441	0.382	0.690	0.129			
	HC	0.715	0.111	–0.423	0.403			
	All	–0.127	0.555	–0.171	0.424			
SiO_2_	OC	–0.498	0.315	–0.780	0.067	0.283	0.493	0.497
	MC	–0.584	0.223	–0.519	0.291			
	IC	–0.391	0.443	–0.507	0.304			
	HC	–0.045	0.932	–0.370	0.470			
	All	–0.242	0.255	–0.206	0.335			
SrO	OC	0.178	0.736	0.025	0.963	**0.000	**3.03E-32	0.470
	MC	–0.255	0.625	0.215	0.682			
	IC	0.086	0.872	–0.052	0.922			
	HC	0.128	0.809	0.023	0.966			
	All	0.027	0.899	0.054	0.804			
MnO	OC	0.637	0.173	–0.473	0.344	0.952	0.692	**0.031
	MC	–0.873	**0.023	0.123	0.817			
	IC	–0.567	0.241	0.137	0.795			
	HC	0.523	0.287	–0.085	0.872			
	All	0.127	0.553	–0.062	0.772			
MgO	OC	0.709	0.115	0.182	0.731	**0.048	**0.000	**0.003
	MC	0.402	0.429	0.621	0.189			
	IC	0.780	0.068	–0.418	0.409			
	HC	0.480	0.335	0.379	0.458			
	All	0.456	**0.025	0.138	0.52			
CaO	OC	0.753	0.084	0.642	0.169	0.089	**0.001	**0.001
	MC	0.137	0.795	–0.555	0.253			
	IC	0.078	0.884	–0.668	0.147			
	HC	0.071	0.894	–0.637	0.174			
	All	0.286	0.176	–0.354	0.090			

** Indicates significant *p*-values. HC: around vascular canals; OC: outer cortical; MC: mid-cortical; IC: inner cortical zones. In the multivariate comparison test, *p*-values are derived from both MANOVA and multivariate comparison testing.

## Results

### Fourier Transform Infrared Spectroscopy Results

**Figure 1.**
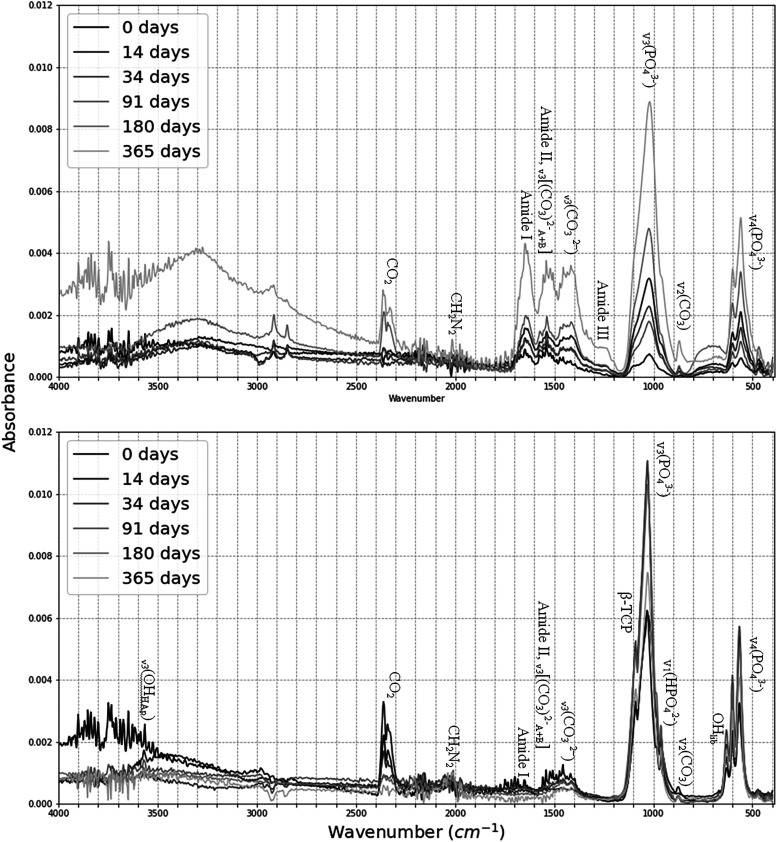
The FT-IR spectra of the means of each PMI group before (upper image) and after (lower image) burning. Labeled are the peak assignments of interest.

The spectra of unburnt and burnt bone were investigated in different regions and their associated changes were noted as a function of length of PMI and burning (Fig. 1; Table II). A change in the intensity of peaks from 0 to 365 days PMI groups were found at several frequencies.

**Table II.** Identified zones of interest and their associated absorption changes on the unburnt and burnt bones.

Wavenumber (cm^–1^)	Functional group	Mode	Bone samples	
			Unburnt	Burnt
3570	Apatitic hydroxyl groups (_versus_OH^–^)	OH stretching^57,93^	N/A	Intensity decreases with longer PMI
3600–2700	Organic fraction and water^94^	C–H and O–H stretching^95^	Convoluted bands, no peaks	N/A
2400–2300	(atmospheric) CO_2_	C≡O stretching^96^	Doublet of bands (traces of CO_2_ from atmosphere)	Doublet of bands (traces of CO_2_ from atmosphere)
2010	Cyanamide (CH_2_*N*_2_)^74,97^	C–N stretching	Weak peak present, highest in 365 day group	Weak peak present, highest in 365 day group
1660	Amide I^57^ (C=O) and water	C–O stretching and H–O–H bending	Decreases with longer PMI	Very weak bands: highest 0 day group, lowest 365 day
1550	Amide II^57^ (N–H)	N–H bending	Decreases with longer PMI	Very weak bands: highest 0 day group, lowest 365 day
1250	Amide III^57^ (N–H)	N–H bending	Shoulder on all groups, except a very weak peak in the 0 day group	N/A
1600–1300	Stretching modes of carbonate ions^94,98^	C–O stretching	Three broad bands with additional peaks; highest: 365 day, lowest: 0 day, mixed in between	Weak bands, highest; 0 day, lowest: 14 day
1415	B type carbonates _v3_(CO_3_^2–^)_B_^98,99^	C–O stretching	Highest of the carbonate peaks, highest in 365 day, lowest in 0 day	Weak peaks: highest in 0 day, lowest in 365 day
1540	A type carbonates _v3_(CO_3_^2–^)_A_^98^	C–O stretching	Overlaps with amide II	Weak peaks: 0 days highest, 365 days lowest
1123	Extra peak, due to appearance of β-TCP^89^	P–O stretching (anti-symmetric (ν3))	No extra peak. Shoulder on the 0 and 91 day groups.	Extra peak around 1080–1090 cm^–1^ on all groups except 34 day group
960–1200	_v1,__v3_ (PO_4_^3–^)^57^	P–O stretching (symmetric (ν1) and anti-symmetric (ν3))	No peaks, shoulders. Most intense: 365 day, least intense: 0 day	Present. Highest intensity: 0, 91, 180 day; weakest: 14 day
872–879	Carbonates (three sub-bands): type A, type B, "labile" _v2_(CO_3_) ^86,94,100^	out-of-plane bending C–O	Present, but as a convolution of the three sub-bands. Highest: 365 day, weakest: 0 day. All the rest are clustered around the same absorbance value.	Present, but as a convolution of the three sub-bands. Decreases with longer PMI. Most intense: 0 day > 14 day. Other groups: clustered around the same lower value
640–620	Apatitic hydroxyl groups^57,101^	(OH) librational	N/A	Present. Intensity of peak: 91 > 34 > 180 > 365 >14 > 0 day
600–500	_v4_(PO_4_^3–^)^98,99^	P–O bending	Two peaks (_v3_ splitting). Most intense: 365 days, least intense: 0 days	Three peaks (_v3_ splitting and OH_lib_ band). _v3_ intensity: 91 > 34 > 180 > 365 > 0 > 14 days
547	Shoulder, due to appearance of β-TCP^89^	P–O bending^98,99^	Present on all. 365 days: strong, 180 days: moderate, 91-, 34-, and 14 days: weak, 0 days: very weak	N/A

The majority of the mean IRSF values for the unburnt bones ranged between 2.88 and 3.30, with the exception of the 365 days bone (5.04) (Table S4). There was an increase in all mean IRSF values post-burning (37.77–57.27%) across all groups, except for the 365 days PMI sample, which decreased by 5.35%. In unburnt bone, the 365 days group’s high IRSF value was significantly different than all other PMI group (Table III). CV values showed very low variation across samples within the same PMI groups. IRSF gives an indication of highest reached temperature and cremation efficiency, due to increased crystallinity values. The sequence of crystallinity of the PMI groups is: 91 > 0 > 180 and 365 > 34 > 14 days. This sequence does not follow the PMI order and it is likely due to the variation in burning conditions (e.g., temperature, duration, and position in the pyre), which are the dominant factors affecting crystallinity.

**Table III.** Linear regression and multivariate comparison test (MCT) on the Fy-IR dataset. Correlation coefficient (*r*) and *p*-value (*p*) for each analyzed FT-IR parameter on the unburnt and burnt bone in the linear regression section. H_0_ = X parameter does not increase with postmortem time period.

FT-IR parameters	Linear Regression				Multivariate comparison of means test		
	Unburnt^a^		Burnt		PMI (PR>F)		State (PR>F)
	r^2^	*p*	r^2^	*p*	Unburnt	Burnt	
IRSF	0.856	**0.029**	0.2	0.704	**0.000002**	**0.014**	**0.001**
C/P	–0.531	0.278	–0.557	0.251	0.505	0.561	**0.001**
BPI	–0.876	**0.022**	–0.619	0.19	**0.006**	0.638	**0.001**
API	N/A	N/A	–0.631	0.179	N/A	0.667	N/A
CO3/P	0.269	0.606	–0.049	0.926	N/A	N/A	N/A
C/C	0.08	0.879	0.096	0.855	**0.034**	**0.003**	**0.024**
CN/P	–0.701	0.12	–0.039	0.941	0.535	0.316	**0.003**
CO/CO3	–0.655	0.158	–0.519	0.29	0.16	**0.003**	**0.005**
PHT	0.679	0.138	–0.159	0.763	**0.032**	0.055	**0.001**
N/P	–0.46	0.357	–0.508	0.303	N/A	N/A	N/A
APR	–0.542	0.267	–0.492	0.321	N/A	N/A	N/A

^a^Items in bold indicate significant *p*-values. The MCT section shows the results of the MANOVA and multivariate comparison testing on the established FT-IR ratios as a function of state (unburnt versus burnt) and time.

The results of the linear regression (Table III) show that there was a strong positive linear correlation between IRSF and time since deposition in unburnt bone (r = 0.856, *p* < 0.029). This increase in structural ordering suggests the early post-mortem recrystallization of bioapatite is already measurable in the first year of PMI. BPI was negatively correlated with PMIs in unburnt bone (*r* = −0.876, *p* < 0.022) (Table III). Although there was no linear change in any of the proposed parameters for PMI estimation in the burnt bone, MANOVA and MCT revealed that IRSF, C/C, and CO/CO_3_ ratios were significantly different in various PMI groups in burnt bone, as well as IRSF, BPI, C/C, and PHT in unburnt bone (Table III).

Unburnt bone at 0 and 91 days PMI displayed an extra shoulder at 1123 cm^–1^, which is assigned for either β-TCP or Mg substitution.^89^ All burnt bone had an extra peak at 1100–1080 cm^–1^, except the 34 day post-mortem group. No burnt bone displayed a shoulder at 547 cm^–1^, but it was a common feature in the unburnt bone, intensity ranging from strong (365 days) to very weak (0 days), which translation band also suggest Mg–OH substitutions.^102^ Mg shows a non-uniform, nonlinear increase in the unburnt bone through the PMI in the EMPA data.

**Figure 2.**
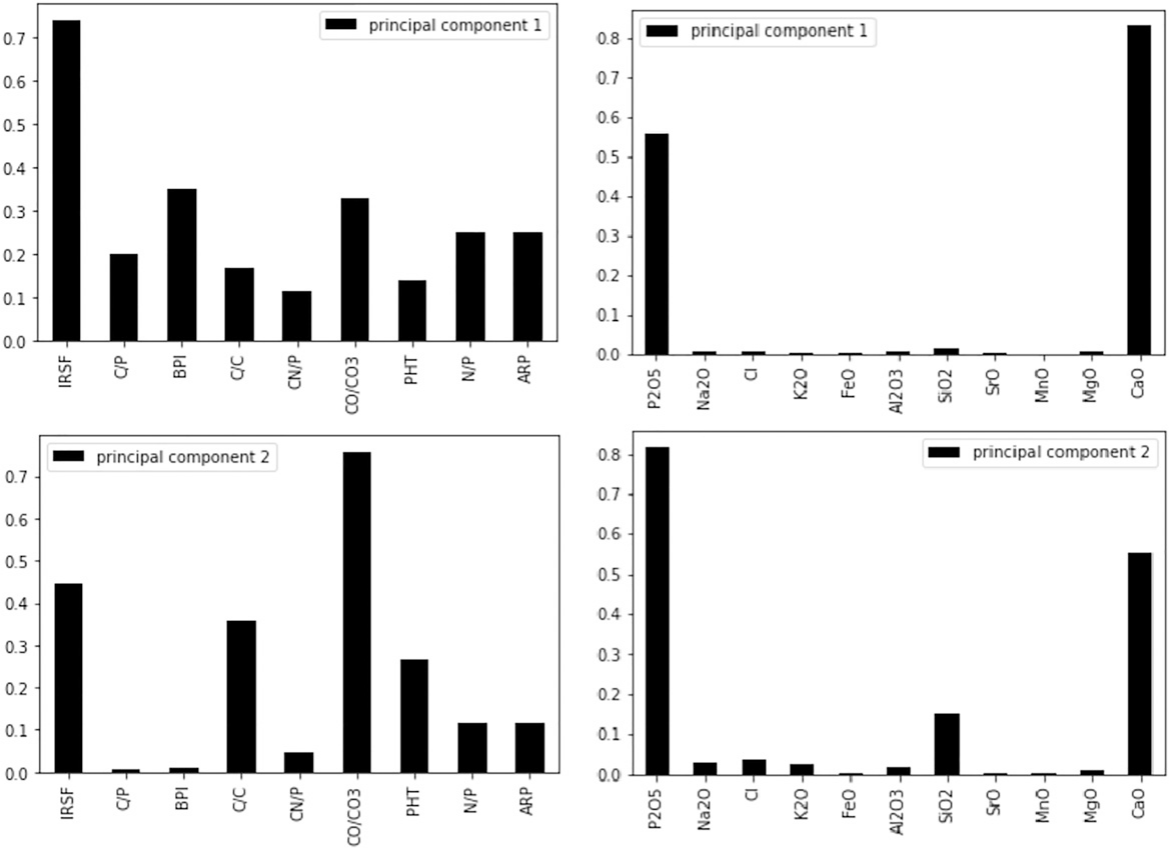
Loadings of the principal component analysis when using both the FT-IR and EMP dataset.

Principal component analysis indicated that the ratios explained 81.67% of the variance in the dataset with the first (PC1) and second (PC2) principal components accounting for 66.67% and 15.00%, respectively. Loadings show that all ratios contributed to PC1, with IRSF (0.74), BPI (0.35), and CO/CO_3_ (0.33) being the largest (Fig. 2), while the main contributors to discrimination by LDA was C/P for burnt bone and PHT and CN/P for unburnt bone (Figure S1, Supplemental Material). Both PCA and LDA differentiated between unburnt and burnt bone, but not between PMI groups.

### WDS-EMP Results

**Figure 3.**
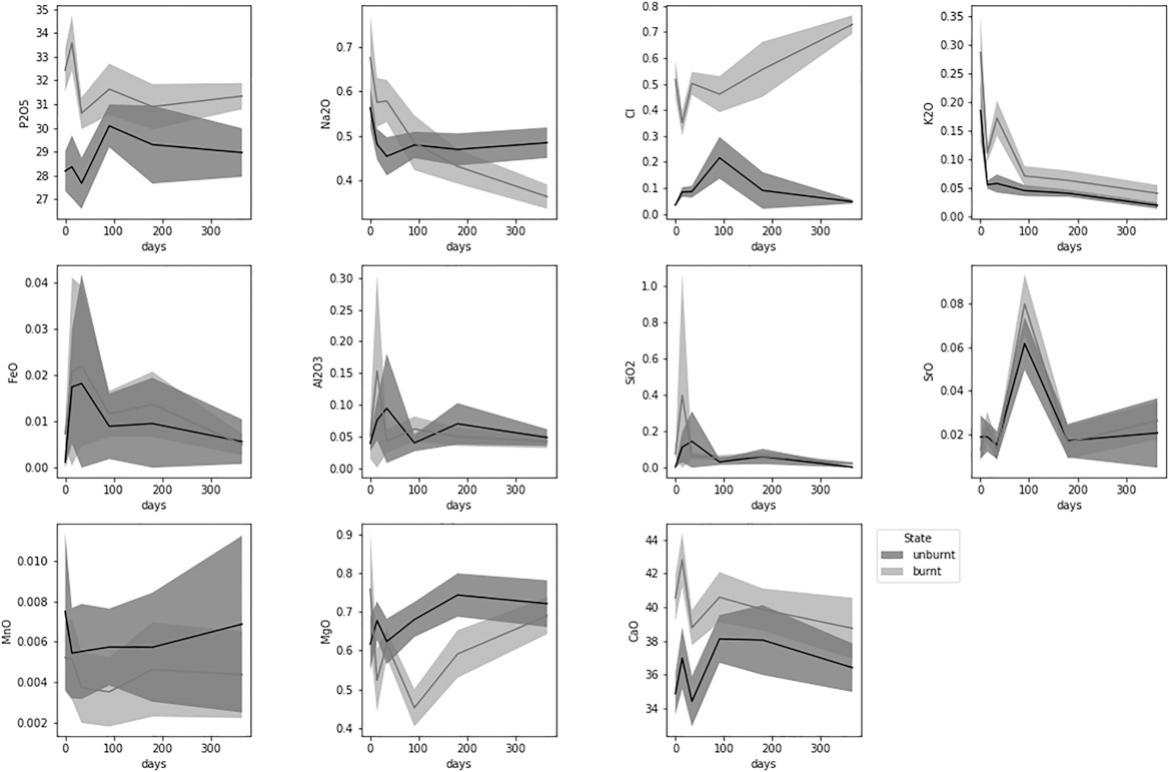
Average elemental concentration (wt %) of the experimental bones using the electron microprobe analysis data. Mean ± 2 standard errors for each measured element as a function of time in unburnt (blue) and burnt (orange) bone.

The WDS-EMP data were analyzed spatially for major and trace elemental concentration (Table S5) of unburnt and burnt bone at different PMIs. Variations in elemental concentrations as a function of time in the experimental bone are visualized in Fig. 3. The most highly variable zones in unburnt bone across all elements were the OC (0.60) and HC (0.50), while IC (0.49) and MC (0.46) were the lowest. The mid-cortical and the inner cortical zones showed the lowest variability across all samples.

**Figure 4.**
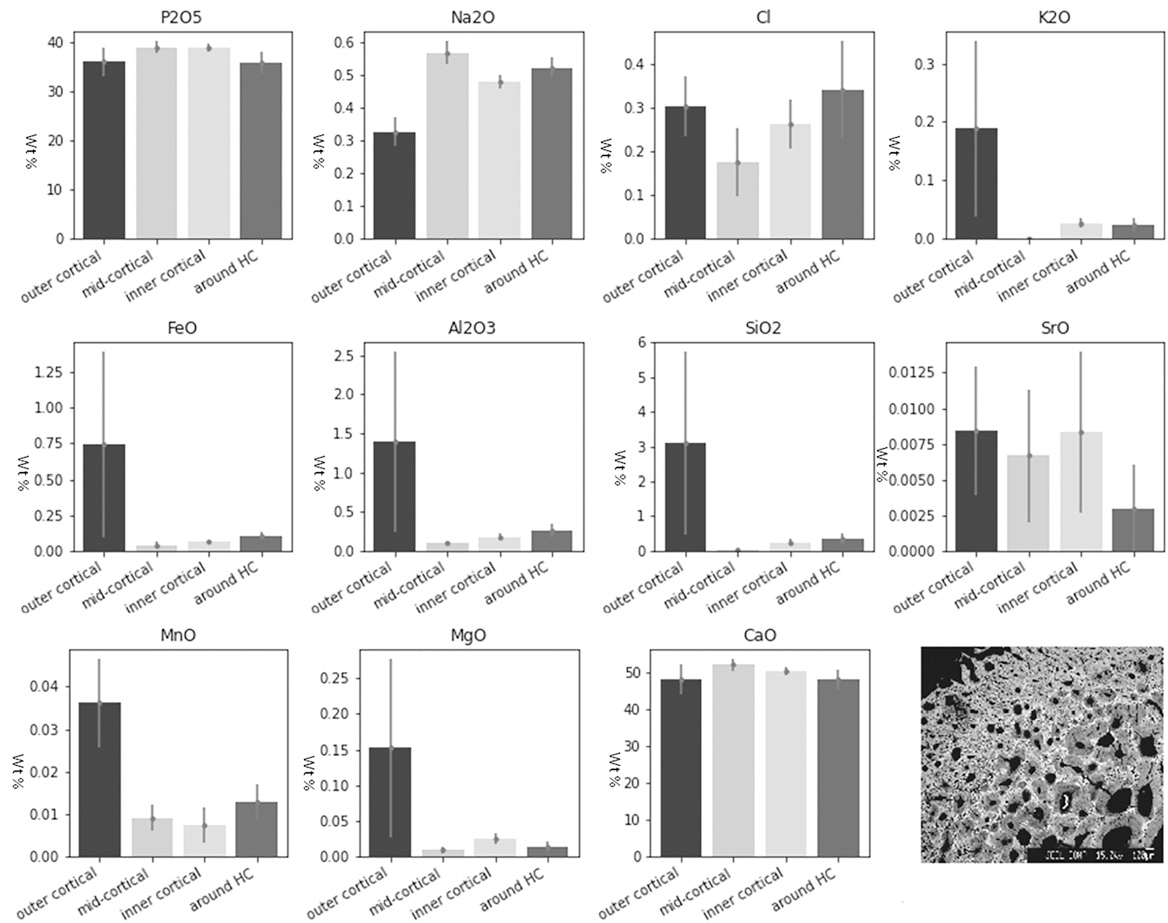
Average elemental concentration (wt %) of the Neolithic cremated human remains for each zone. Mean ±2 standard errors for each measured major and trace element in archaeological cremated bone. The backscattered electron microscopy (BSEM) image represents an example of a bone cross section.

In the archaeological cremated bone (KILT) P and Ca remained relatively stable in all zones, while K, Fe, Al, Si, Sr, Mn, and Mg display very high values in the outer cortical layers, suggesting diffusion of elements from the soil (Fig. 4). The highest CV scores were recorded for Sr, especially in the outer cortical layers.

**Figure 5.**
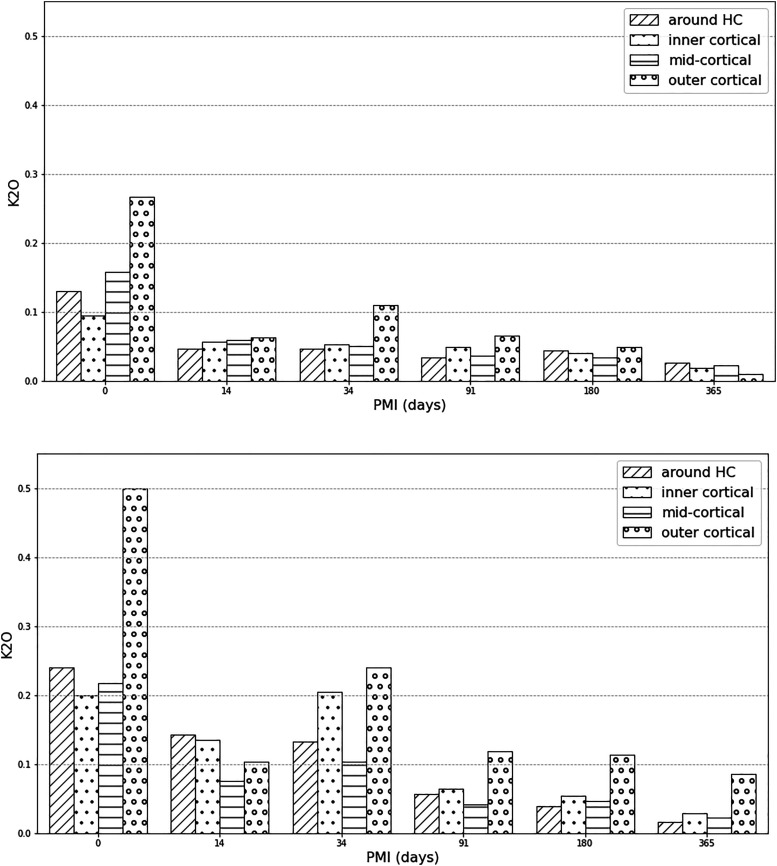
Potassium wt% content in the experimental unburnt (upper) and burnt (lower) bone by zone.

Linear regression analysis (Table IV) shows that only K, Mn, and Mg in unburnt bone and Na, Cl, and K in burnt bone changed in a linear fashion with time. K was the only element that decreased both in the unburnt and burnt bone sections overall, and separately in the IC and HC zones (Fig. 5). There were no significant trends in the Ca/P ratios and all zones presented similar values (between 1.23 and 1.29) while the archaeological samples were insignificantly higher (1.35).

**Figure 6.**
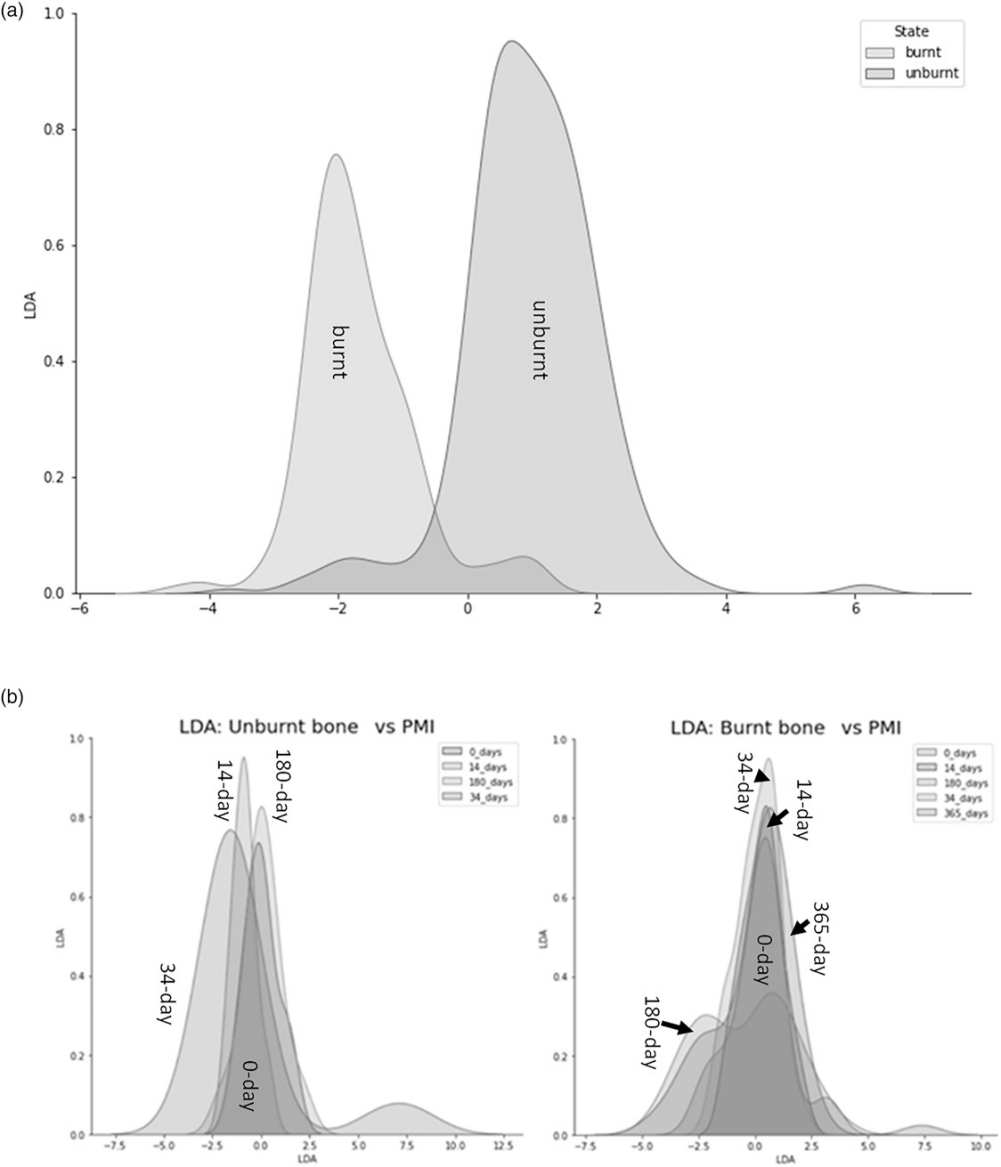
Linear discriminant analysis (LDA) on the electron microprobe analysis dataset. (a) Separation of unburnt and burnt bone by LDA. Contributions: Al_2_O_3_: 0.244, CaO: -6.882, Cl: 3.216, FeO: 0.07, K_2_O: 0.607, MgO: –1.036, MnO: –0.056, Na_2_O: –0.545, P_2_O5: –3.97, SiO_2_: –0.902, SrO: 0.199, Total: 13.08. (b) Clusters created by LDA of the (left) unburnt and (right) burnt PMI groups.

The PCA showed that the first principal component (PC1) explained 93.49% of the variance and the second principal component (PC2) explained 4.19%, with the key discriminators being Ca and P (Fig. 2). LDA was used to reduce the features (elements), classify samples, and subsequently visualizing the classification according to the defined categories (state, PMI, and zone). When the data were transformed into a two-feature (unburnt versus burnt) linear discriminant dataset, the two states were clearly differentiated (Fig. 6a). LDA did not clearly differentiate between the PMI groups, with slightly more separation of PMIs in unburnt bone (Fig. 6b). MANOVA and MCT showed that P, Na, Cl, K, Mn, Mg, and Ca were significantly associated with burning, while Fe, Si, Sr, and Al were not (Table IV). An overall significant difference was observed between PMI groups in Cl, K, Sr, and Mg levels in unburnt bone, and in P, Na, Cl, K, Sr, Mg, and Ca values in burnt bone (Table IV). The fresh group differed significantly in K values from all other PMI groups in both unburnt and burnt bone by MANOVA and MCT.

## Discussion

This study on the early diagenesis and burning of bioapatite has shown that both structural and chemical changes are observable after only one-year PMI. Potassium elemental concentration decreased with PMI and survived burning, making it the best discriminator between bone burnt fresh and partially decomposed. This decrease compared to experimental bone suggests that endogenous K had decayed prior to cremation in the Neolithic bone, thus the bodies might have been left to decompose prior to further funerary treatment. While decrease in carbonates and increase in crystallinity were documented in only one year of surface exposure, these were masked by burning. Ca, P, Fe, Al, Si, and Sr did not significantly change with heat-treatment and Fe, Al, Si, and Sr were not associated with time since deposition either.

Crystallinity increases in a linear fashion with PMI in unburnt bone and burnt bone shows higher crystallinity compared to unburnt bone. This agrees with the literature (e.g., Thompson et al.^67^ and Snoeck et al.^74^), except the 365 day burnt group where results show a slight decrease, which may be the result of the already higher crystallinity of the bone or small-scale temperature variation across individual bones. The lack of linear increase in burnt bone crystallinity with time suggests that burning overrides the increase in IRSF due to early diagenesis in the first six months of the PMI. As crystallinity increased, carbonate content decreased in a linear fashion in the unburnt bone, in line with other studies.^24,103^ Keenan and Engel^24^ documented significant carbonate loss at three years PMI, with insignificant decrease after one year but increase compared to that value in two years, while here A and B carbonates to phosphate ratios showed a significant linear decrease throughout only a year PMI. However, B type carbonates only significantly changed prior to three months PMI compared to the 365 days group. Burning, again, obscured the effects of early diagenesis of carbonate loss.

β-TCP signals the thermal decomposition of the apatite phase and should be detectable in bones burnt at >700 °C,^69,70,104^ which was reached in this study for a short duration. There is an extra peak at ∼1100–1080 cm^–1^ on all burnt bone spectra, which might be due to a shift in the spectra and might still represent β-TCP. However, the lack of a shoulder at 547 cm^–1^ suggests otherwise. Surprisingly, all unburnt bone displayed a shoulder at 547 cm^–1^. These shoulders cannot be due to β-TCP in unburnt bone but are probably due Mg substitutions for Ca.^89^ However, there were no outstanding elevated levels of Mg in the unburnt bone (unburnt mean Mg levels ranged between 0.01 and 0.82 wt%). Mg values in all zones are slightly higher in the 180- and 365 days groups than those with shorter PMIs, suggesting a non-uniform, nonlinear increase. However, Mg values were highly variable in burnt bone. With a melting point of 650 °C, this might be due to the burning efficiency of the different fire events. Therefore, β-TCP as a thermal decomposition product might not have occurred, but Mg ions might have increased despite its reduced competitiveness in substituting due to the crystal lattices’ more accommodating nature for sodium and potassium.^105^ Another possibility is that β-TCP did appear as a thermal decomposition product, but post-cooling it was not possible to detect, as it is not thermally stable.^106^

As the bones were burnt in an outdoor fire, burning efficiency was variable. Burning efficiency could be estimated for the different PMI groups from the N/P ratios and IRSF. The sequence from highly efficient to less efficient from N/P was 91- and 180 days > 365 days > 34 days > 0 days > 14 days, while from IRSF data it was 91 days > 0 days > 180- and 365 days > 34 days > 14 days. The differing placement of the 0 days group in the two sequences might be due to the placement of the different fat-content of the individual fresh legs, since fat acts as a fuel during burning, increasing temperature when more fat is present and resulting in higher crystallinity. MANOVA results confirmed that all FT-IR ratios designed to assess cremation efficiency^19,67,81^ differ significantly in unburnt and burnt bone. In both cases, the 91 days group was the most efficiently cremated and the 14 days group the least. This might explain why only the 14 days and the 0 days groups showed significantly different values in P in burnt bone. Ca and P, the two major elements in bone, were otherwise insignificant in both the unburnt and burnt groups. Sr was significantly different in the most efficiently burnt group (91 days) than in all others, but this difference was present in its unburnt state as well; thus, it was not due to burning.

Other than Mn, the increase in all other element’s atomic percentage is higher in burnt 0 days (fresh) bone than in unburnt fresh bone. However, it appears this is due to the decrease in organic content and thus does not essentially indicate a real increase in the mineral phase. Ca, P, Fe, Al, Si, and Sr changes through the PMI stages in the same fashion in unburnt and burnt bone, meaning they are not highly affected by burning at ≤750 °C and their amount in burnt bone is representative of their pre-burnt levels. The results for Sr agree with those of Snoeck et al.,^74^ who did not find changes in Sr isotopic signals following experimental cremation. Fe showed an initial increase at 14 days PMI and a sharp decrease thereafter, which is due to soft tissue putrefaction and aligns with the literature.^25^

Only three elements (K, Na, Cl) were found to be affected by time since deposition in the first year PMI. Potassium and sodium are associated with moisture in bone and their decrease signifies dehydration.^25^ Here, potassium was the most useful element in differentiating between fresh (0 days) and all other PMI groups in unburnt bone. In addition, K showed an overall linear decrease in both unburnt and burnt bone area for all zones combined. The unburnt group’s potassium values suggest rapid depletion of K in two weeks (from mean 0.16 wt% to mean 0.06 wt%), after which depletion slowed down. Environmental factors might have altered the rate of depletion in the OC and IC zones. After 34 days, bone had a higher K-value in the OC zones, which coincides with the time at which the specimens started to be noticeably partially submerged into the soil. The burnt experimental bone always had higher K-levels than unburnt but followed the same pattern of depletion. Although KOH has a relatively low melting point (360 °C), it was found in apatitic aorta deposits burnt at 720 °C,^107^ suggesting that it is bound to the crystal. K was previously found to be localized in the removed organic bone material^108^ and was suggested to be present mainly in a labile state, meaning it is localized on the mineral crystal surface with low migration activity.^107^ However, the latter study’s limitations included a more limited detection limit of EDS and the inability to evaluate the contributions to the labile fraction corresponding to surface-localized water-soluble compounds related to the organic matrix. Since organic material was removed by burning in the present study’s bones, meaning that the oxidation of carbon was complete (assessed by the white color of calcined bones without black ash/carbon left behind), but K was still detectable by EMPA, it is possible that K integrates into the crystal lattice. The 0 days burnt bioapatite samples always had K-values ≥0.20 wt%, while after 14 days this decreased to <0.14 wt% in all areas. The OC zones also showed depletion but at a slower rate than other areas. After three months, all K concentrations at IC, MC, and HC zones were ≤0.06 wt% and <12 wt% at OC areas. Thus, K-levels of <0.07 ± 0.001 wt% in the inner and mid-cortical zones, and around vascular canals of burnt bone suggest that the corpse was not burnt immediately after death, but after around three months. The outer cortical zones might show higher K concentrations due to uptake of potassium from the soil. It has to be noted that these bones were surface exposed, and that dehydration would be expected to be slower had they been buried. In the Neolithic human bone fragments, endogenous K was below detection limit in the mid-cortical bone sections. Exogenous K (mean 0.08 wt%) from the soil might have diffused into the OC layers and to a lesser degree from the IC end and through the HC zones. Since bioapatite is less susceptible to changes post-burning, due to increased crystallinity,^42,73,74,109^ this might mean that potassium essentially depleted in the Neolithic samples prior to cremation. Therefore, these bodies might have been left to decompose prior to cremation, to a degree that endogenous potassium was no longer present. Based on the experimental results of this study, in which K-values were never below the detection point at any zone, the postmortem interval prior to cremation in the archaeological bone can be suggested to have been >1 year.

Sodium is also associated with extracellular fluid in the body.^25^ The 0 days burnt group showed higher values than the unburnt group, which agrees with Ellingham et al.^48^ In the experimental burnt samples there was a slight decrease between the 0 days (0.54 wt%) and 14 days (0.46 wt%) groups, after which it remained stable (0.45–0.48 wt%). Previously, Na was shown to have a tendency of mass transfer, leaving the crystals at 720 °C and increasing at 760 °C, which was suggested to be due to the reversible exchange of CO_3_^2–^.^107^ Here, Na showed a linear decrease with time in heated bones. The decrease of Na in the EMPA data is confirmed and supported by the decrease of CO_3_ ions in the FT-IR data.^110^ The initial value was 54% lower than in modern human bone reported by Elliott.^28^ This discrepancy might be due to inter-species differences in calcium-substituting ions, such as Na, but studies on them are sparse.^111^ The Na value of 0.47 wt% for the Neolithic bone is consistent with that of unburnt archaeological bone reported in the literature.^112^ The burnt bone demonstrated that MC, IC, and HC zones had similar Na values, while OC zone was depleted. Normally, Na leaches out of the layers of bone closest to the environment over time,^112,113^ but these bones were broken. Nevertheless, it seems to explain why the experimental burnt bone had higher Na values in the OC zones one year post-mortem than the archaeological samples.

Chlorine values in burnt bone behaved almost inversely proportional to unburnt bone over time. Cl-enriched phases might appear in early diagenetic bone through the incorporation of Cl^–^ facilitated by the protonation of hydroxyl ions,^35^ and/or by burning above 600 °C,^114^ up to <800 °C in defleshed bone and <900 °C in fleshed bone.^48^ OH sites in bone can be substituted with Cl.^110^ An OH–Cl peak was observed in the FT-IR spectra of the burnt bones in all PMI groups, suggesting that Cl substitution for OH started in these bones. Our results are supported by the agreement between the EMPA and the FT-IR results and previous studies’ results showing Cl incorporation when HAp is heated above 600 °C.^114^

Principal component analysis and LDA formed clearly separate clusters of unburnt and burnt groups but were unable to distinguish between PMI groups. C/P, PHT and CN/P were the main discriminating ratios between the state of bone. Thus, FT-IR is a useful technique in differentiating between unburnt and burnt bone, but it cannot detect early diagenesis up to one year postmortem in burnt bone. The main discriminating chemical elements between unburnt and burnt bone were Ca and Cl by LDA, and Ca and P using PCA.

The Ca/P molar ratios remained fundamentally constant not only during soft tissue putrefaction,^47^ but also at one year PMI. The fact that this ratio did not present significant alterations with burning makes it a characteristic signature of bone, further supported by the similar Ca/P ratios of the archaeological and experimental burnt bone. Ratios were much lower than for stoichiometric HAp, due to the lattice housing carbonates and other elements.^28^ Both Ca and P remained stable during the entire PMI. For Ca, this agrees with Reidsma et al.,^26^ but in contrast to findings by Subirà and Malgosa^58^ and McKinnon et al.^71^ Although a decrease in C/P ratio had been documented after one year of burial,^24^ here C/P ratios (from the FT-IR dataset) did not change significantly with PMI, suggesting a stable ratio of carbonate to phosphate during the studied PMI in the same unburnt or burnt groups. The discrepancy with Keenan and Engel’s^24^ results might be due to the fact that their experimental alligator bones had evidence of microbial bioerosion, while this was not present in this study’s bones.^22^ Nevertheless, time since deposition does not seem to be the driving cause of C/P variation.

The outer cortical layer’s highest variation in elements in unburnt bone demonstrates that exogenous elements diffuse into the bone in less than one year and endogenous elements are lost. However, zones around the vascular canals show the most varied elemental chemistry once bone is burnt. The high variability in concentration around osteons aligns with a microsampling study of unburnt bone by Scharlotta et al.^115^ which found the presence of diagenetically unaltered zones further from the osteons. K, Si, Al, and Fe were barely detectable in the mid-cortical section in the archaeological bone, while the levels of these elements were high in the OC layers. However, in the experimental bone, the MC section did exhibit changes in elemental concentration with time. In other words, the mid-cortical section is more resistant to diagenesis than other areas of the bone, but it is also taphonomically altered, evidenced in <1 year postmortem.

## Conclusion

Fire drastically changes the morphological and chemical profile of bone, making it notoriously difficult to derive information about circumstances surrounding death and deposition or burial of an individual. This includes how cultures treated their dead prior to and during cremation, and how cause and manner of death are identified after burning in forensic cases. Our investigation indicates that crystallinity increases in unburnt bone are detectable during the first year postmortem. B-type carbonate content decreased rapidly in the first three months PMI in unburnt bone, after which it slowed down. Both changes are masked by burning. The thermal decomposition of the apatite phase did not occur post-burning from the lack of β-TCP signals in the IR spectra. The presence of these signals in unburnt bone suggests Mg substitutions, supported by the non-uniform, nonlinear increase of Mg values with time from the EMPA data.

Major (Ca, P) and some trace elements (Fe, Al, Si, and Sr) were not significantly associated with thermal treatment, while Fe, Al, Si, and Sr were not associated with the PMI. K, Na, Mg, and Cl were the only elements associated with time since deposition in the experimental bone. Potassium showed a potential to estimate the late postmortem interval/early diagenesis in both unburnt and burnt bone in the first year postmortem. Potassium rapidly depleted in bone after two weeks and continued to decrease over one year PMI. It is proposed here that if the K-values are under 0.07 ± 0.01 wt% in the inner and mid-cortical zones of burnt bone then they were not burnt subsequently after death, but after at least three months decomposition in a surface exposed environment, or after a few months/years if buried, since burial slows down dehydration. Since K was present in calcined bones, it is proposed here that it might not exclusively be present in the organic phase of bones. In the Neolithic human cremated bones endogenous potassium is below detection limits. Therefore, if the bioapatite crystal structure truly become thermodynamically stable after burning at high temperatures as previous studies indicate,^42,73,74^ then it suggests that cremation of these corpses was delayed by at least some weeks following death.

Sodium, which is also associated with the extracellular fluid, rapidly decreased in 2 weeks in both unburnt and burnt bone, after which it remained stable in the former, but continued to decrease at a higher rate in the latter. The decrease of Na in the burnt bones was also confirmed by the decrease of CO_3_ ions. The increase of Cl in the burnt bones with time was confirmed by the EMPA data and the start of the Cl substitutions for OH by the OH–Cl peaks in the FT-IR data.

Principal component analysis and LDA were moderately successful at differentiating between unburnt and burnt bone, but not between PMIs of up to one year. C/P, PHT, and CN/P from the FT-IR and Ca, Cl, and P from the EMPA were the best discriminators for unburnt versus burnt state of bone. This study demonstrates that Ca/P molar ratios do not change significantly due to PMI or burning in the experimental and only insignificantly change in the archaeological bone. However, C/P ratios might be dependent on the presence of microbial bioerosion rather than the time since deposition. Elemental chemistry analysis demonstrated that the mid-cortical zone is the least affected by contamination in both a modern and archaeological timescale.

This study has relevance for the assessment of postmortem interval, deposition environment, and time of burning in analysis of archaeological cremated bones and of burnt human remains in forensic cases.

## Supplemental Material

Supplemental Material - Physicochemical Changes in Bone Bioapatite During the Late Postmortem Interval Pre- and Post-Burning Click here for additional data file. Supplemental Material for Physicochemical Changes in Bone Bioapatite During the Late Postmortem Interval Pre- and Post-Burning by Emese I. Végh, Nicholas Márquez-Grant, and Rick J. Schulting in Applied Spectroscopy
