# Supplementary material for: Physicochemical Changes in Bone Bioapatite During the Late Postmortem Interval Pre- and Post-Burning
Source: Appl Spectrosc. 2022 Jun 17;76(9):1080–99. doi: 10.1177/00037028221085600 (PMC9490440; doi:10.1177/00037028221085600)
Supplement: Supplemental Material - Physicochemical Changes in Bone Bioapatite During the Late Postmortem Interval Pre- and Post-Burning [file sj-pdf-1-asp-10.1177_00037028221085600.pdf]

Supplemental Material

Physicochemical Changes in Bone Bioapatite During the Late Postmortem Interval Pre- and Post-Burning

Emese I. VÉGH[1],* Nicholas MÁRQUEZ-GRANT[2], and Rick J. SCHULTING[1]
[1]Research Laboratory for Archaeology and the History of Art, University of Oxford, Dyson Perrins Building, South Parks Road, Oxford OX1 3QY, UK
[2]Cranfield Forensic Institute, Cranfield University, Defence Academy of the United Kingdom, College Rd, Cranfield, Wharley End, Bedford MK43 0AL, UK
* Corresponding author email: emese.vegh@wolfson.ox.ac.uk

Table S1. The reported elemental concentration of bone ±1% variability due to diet, age, and anatomical variation (after Elliott,[28] p. 430).

**Component**	**Bone (wt%)**
Ca	36.6
P	17.1
CO_2	4.8
Na	1
K	0.07
Mg	0.6
Sr	0.05
Cl	0.1
F	0.1
Ca/P molar	1.65

Table S2. Documented differences in elemental concentration due to burning in bone in the literature.

**Heat-Induced Elemental Changes in Bioapatite**			
**Element(s)**	**Noted Differences**	**Notes**	**Citations**
Sr, Mg, Zn, Cu, Ba, V, Mn,	Lower in archaeological calcined bones than in unburnt bones.	Potential contamination from the soil in unburnt bones.	63

Pb			
Sr	Stable	Calcined bones do not exhibit uptake or loss of Sr, making them a reliable substrate for strontium isotope analysis.	114
Ca	Increase	Not statistically significant	81
	Stable	Together with P	43
	Decrease	Different between temperature groups, but not between archaeological and modern bones.	99
Mg, F	Decrease	At >500°C and only in specific surface zones	97
Fe	Increase	Uptake in archaeological charred enamel suggested to be caused by pores and fissures from burning.	78

Table S3. Measured IR ratios and their assignments from the literature.

**Wavenumber (cm^{-1})**	**Notation**	**Assignment**	**Information retrieved**
(565 + 603)/595	IRSF	Crystallinity Index or Splitting Factor, splitting between the $_{v3}PO_4$ domain	Reveals the structural order of the crystals of the inorganic portion of bones [4,7,93,107,129,148,149]
1660/1035	APR	Amide to phosphate ratio	Collagen to phosphate ratio[150]
1415/1035	C/P	Carbonate (CO_3) to phosphate (P) ratio	Type B carbonate content of the sample [112]
1415/603	BPI	Type B carbonate to phosphate ratio	Amount of type B carbonate

1540/603	API	Type A carbonate to Phosphate	Amount of type A carbonate (only calculated from burnt bone due to overlap with amide II)
1450/1415	C/C	type B to type A carbonate	Amount of type B carbonate compared to type A carbonate
2010/1035	CN/P	Cyanamide (CN_2^{2-}) to phosphate	Cyanamide presence[8]
1650/1415	CO/CO3	Carbonyl to Carbonate ratio	Organic to mineral ratio
625/610	PHT	Phosphate high temperature	Bones burnt >700^{0}C[95]
1660/1035	N/P	Amide I to Phosphate	Organic to mineral ratio

Table S4. The mean IRSF values for the unburnt and burnt groups at different PMIs.

**Descriptive Statistics of the IRSF Measurements**										
**PMI (days)**	**Sample**	**Unburnt**	**Burnt**	**Mean Unburnt**	**Mean Burnt**	**Mean % increase**	**Std Unburnt**	**Std burnt**	**CV unburnt**	**CV burnt**
0	WSFresh	3.16	5.16	3.08	5.03	52.42	0.44	0.19	0.13	0.04
	SWF2FR3	2.99	5.04							
	SWF2FR4	N/A	4.93							
	WS4_FR	N/A	5.24							
	WS5_FR	N/A	4.76							
14	WSF2D2W1	2.49	3.58	2.88	4.10	42.36	0.23	0.75	0.08	0.18
	WSF2D2W2	2.91	5.08							
	WSF2D2W3	3.06	3.2							
	WSF2D2W4	3.01	4.56							
	WSF2D2W5	2.95	4.07							

34	WSF3D1M1	3.18	4.71	3.23	4.45	37.77	0.21	0.27	0.06	0.06
	WSF3D1M2	3.53	4.29							
	WSF3D1M3	2.98	4.68							
	WSF3D1M4	3.31	4.06							
	WSF3D1M5	3.14	4.49							
91	WSF3D3M1	3.48	5.04	3.31	5.19	57.27	0.12	0.37	0.03	0.07
	WSF3D3M2	3.28	5.57							
	WSF3D3M3	3.22	5.41							
	WSF3D3M4	3.25	4.75							
180	WSF5D6M1	2.96	5.26	3.07	4.77	0.55	0.07	0.34	0.03	0.07
	WSF5D6M2	3.03	4.36							
	WSF5D6M3	3.16	4.55							
	WSF5D6M4	3.12	4.83							
	WSF5D6M5	3.1	4.85							
365	SWF5D1Y1	5.04	4.77	5.04	4.77	-5.35	0.00	0.00	0.00	0.00

Figure S1. Linear discriminant analysis (LDA) on the FT-IR dataset to discriminate between states (unburnt versus burnt) bone. Contributions: ARP: -3.806, BPI: 3.204, C:C: -3.97, C:P: -21.982, CN:P: 14.449, CO:CO3: -1.345, IRSF: 4.651, N:P: -3.806, PHT: 19.552.

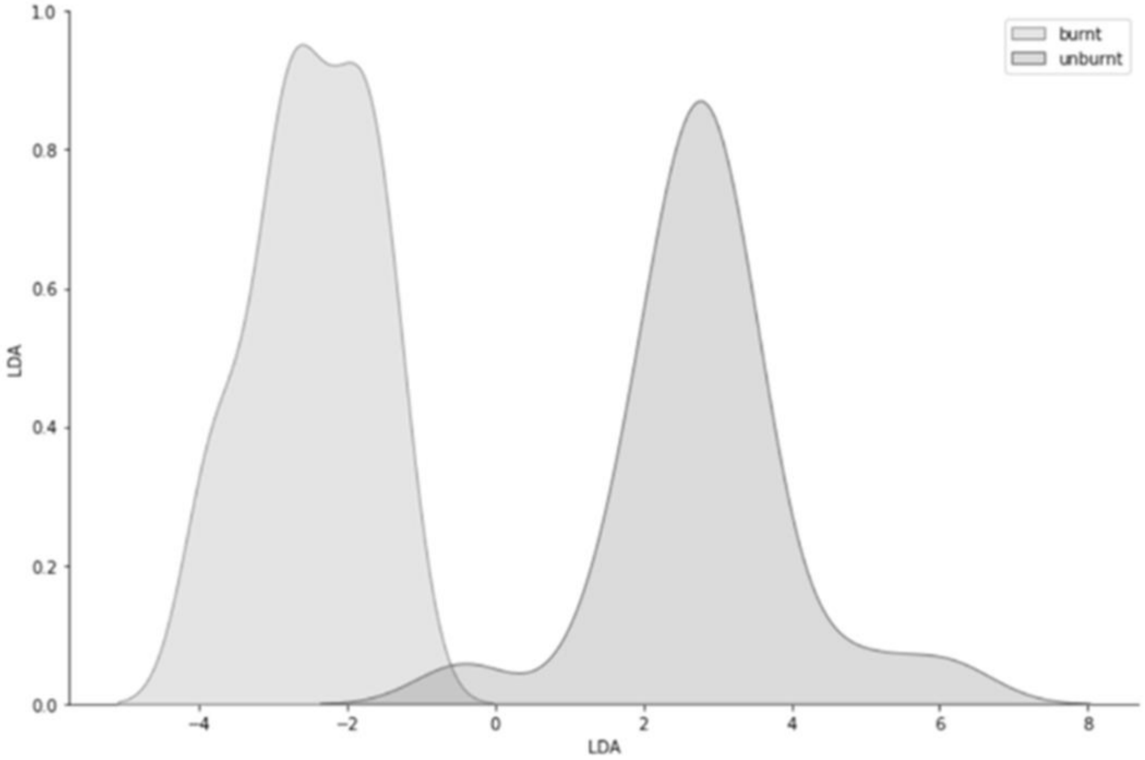

Table S5. Mean major and trace elements chemistry, coefficient of variation (CV) of the unburnt (U) and burnt (B) bone samples with different PMIs. N= analysis per specimen.

PMI	State	Zone	N	**P2O5**		**Na2O**		**Cl**		**K2O**		**FeO**		**Al2O3**		**SiO2**		**SrO**		**MnO**		**MgO**		**CaO**	
				mean	CV	mean	CV	mean	CV	mean	CV	mean	CV	mean	CV	mean	CV	mean	CV	mean	CV	mean	CV	mean	CV
0 days	U	HC	3	27.87	0.07	0.51	0.18	0.03	0.00	0.13	0.00	0.00	0.00	0.03	1.00	0.00	0.00	0.02	1.00	0.01	1.00	0.56	0.16	33.81	0.09
		IC	2	27.24	0.02	0.46	0.02	0.04	0.00	0.10	0.10	0.00	0.00	0.05	0.20	0.00	0.00	0.00	0.00	0.01	1.00	0.54	0.20	34.03	0.04
		MC	5	29.34	0.05	0.57	0.11	0.03	0.33	0.16	0.50	0.00	0.00	0.05	0.40	0.01	3.00	0.02	1.00	B.D.	0.00	0.68	0.16	37.31	0.05
		OC	6	27.69	0.06	0.62	0.16	0.04	0.25	0.27	0.44	B.D.	0.00	0.04	0.75	0.00	0.00	0.02	1.00	B.D.	0.00	0.62	0.31	33.67	0.06
		Total	16	**28.03**	0.05	**0.54**	0.11	**0.03**	0.33	**0.16**	0.31	**0.00**	0.00	**0.04**	0.50	**0.00**	0.00	**0.02**	1.00	**0.01**	1.00	**0.60**	0.20	**34.70**	0.06
	B	HC	14	32.44	0.12	0.86	0.65	0.35	0.46	0.24	0.75	B.D.	0.00	0.10	3.20	0.08	0.88	0.01	2.00	B.D.	0.00	0.75	0.71	41.34	0.12
		IC	22	32.79	0.16	0.57	0.40	0.56	0.80	0.20	0.55	B.D.	0.00	0.04	2.25	0.06	0.83	0.01	2.00	B.D.	0.00	0.85	1.20	42.07	0.17
		MC	18	32.15	0.06	0.58	0.41	0.51	0.33	0.22	0.50	B.D.	0.00	0.02	1.50	0.09	1.33	0.02	1.00	B.D.	0.00	0.59	0.17	40.40	0.05
		OC	1	32.28	0.11	0.77	0.66	0.60	0.4	0.5	0.9	B.	0.0	0.0	0.8	0.0	1.0	B.	0.0	B.	0.0	0.8	0.1	38.2	0.1

			8						7	0	0	D.	0	5	0	7	0	D.	0	D.	0	2	3	0	8
		Total	72	**32.42**	0.11	**0.69**	0.55	**0.51**	0.51	**0.29**	0.72	**N/A**	0.00	**0.05**	2.40	**0.08**	1.00	**0.01**	2.00	**N/A**	0.00	**0.75**	0.59	**40.50**	0.13
14 days	U	HC	12	29.49	0.12	0.46	0.13	0.08	0.75	0.05	0.40	B.D.	0.00	0.05	1.20	0.02	1.50	0.02	1.50	B.D.	0.00	0.72	0.18	38.31	0.11
		IC	16	28.18	0.08	0.47	0.34	0.09	0.67	0.06	0.33	0.02	3.00	0.10	1.40	0.08	1.63	0.01	3.00	B.D.	0.00	0.63	0.29	36.94	0.11
		MC	18	30.95	0.10	0.56	0.13	0.05	0.40	0.06	0.50	B.D.	0.00	0.03	1.33	0.02	1.50	0.03	0.67	B.D.	0.00	0.78	0.12	40.35	0.12
		OC	7	20.16	0.32	0.33	0.33	0.14	0.93	0.06	0.50	0.08	0.75	0.16	1.25	0.59	1.14	B.D.	0.00	B.D.	0.00	0.45	0.47	26.11	0.31
		Total	53	**27.19**	0.14	**0.46**	0.22	**0.09**	0.78	**0.06**	0.50	**0.05**	0.60	**0.09**	1.22	**0.18**	1.17	**0.02**	1.00	**N/A**	0.00	**0.65**	0.23	**35.43**	0.15
	B	HC	15	31.78	0.19	0.62	0.53	0.33	0.48	0.14	0.64	0.06	3.00	0.58	2.28	1.67	3.60	0.02	1.50	B.D.	0.00	0.61	0.85	40.83	0.17
		IC	17	35.60	0.13	0.56	0.30	0.33	0.82	0.13	0.54	0.01	2.00	0.04	0.50	0.06	1.17	0.03	1.00	B.D.	0.00	0.53	0.53	45.66	0.15
		MC	21	34.41	0.13	0.57	0.42	0.30	0.57	0.08	0.50	B.D.	0.00	0.03	1.00	0.02	1.50	0.02	1.00	B.D.	0.00	0.43	0.74	44.37	0.13
		OC	17	32.15	0.11	0.55	0.31	0.46	0.43	0.10	0.50	0.02	1.50	0.03	1.00	0.09	1.00	0.02	1.50	B.D.	0.00	0.56	0.36	39.77	0.14

		Total	70	**33.49**	0.14	**0.58**	0.40	**0.35**	0.57	**0.11**	0.55	**0.03**	2.00	**0.17**	2.06	**0.46**	3.37	**0.02**	1.50	**N/A**	0.00	**0.53**	0.62	**42.66**	0.15
34 days	U	HC	10	28.75	0.08	0.49	0.27	0.08	0.38	0.05	0.60	B.D.	0.00	0.04	0.75	0.05	1.00	0.02	1.00	B.D.	0.00	0.71	0.15	36.61	0.10
		IC	11	26.76	0.12	0.43	0.42	0.08	0.38	0.05	0.60	B.D.	0.00	0.07	0.57	0.14	1.14	0.01	2.00	B.D.	0.00	0.52	0.46	32.67	0.12
		MC	12	28.53	0.09	0.47	0.15	0.07	0.43	0.05	0.40	B.D.	0.00	0.05	1.00	0.02	2.00	0.01	2.00	B.D.	0.00	0.69	0.16	35.43	0.11
		OC	5	25.41	0.23	0.41	0.37	0.17	1.00	0.11	1.00	0.11	1.73	0.38	1.89	0.65	2.17	0.02	1.50	0.01	0.00	0.53	0.32	31.48	0.24
		Total	38	**27.36**	0.13	**0.45**	0.29	**0.10**	0.60	**0.07**	0.71	**0.11**	0.45	**0.13**	1.62	**0.22**	1.86	**0.02**	1.00	**0.01**	1.00	**0.61**	0.26	**34.05**	0.14
	B	HC	13	30.65	0.12	0.58	0.22	0.47	0.40	0.13	0.77	B.D.	0.00	0.07	1.86	0.06	1.00	0.01	2.00	B.D.	0.00	0.62	0.29	39.13	0.14
		IC	16	30.95	0.11	0.62	0.42	0.51	0.20	0.20	0.75	B.D.	0.00	0.03	1.33	0.06	0.83	0.02	1.00	B.D.	0.00	0.59	0.27	38.99	0.12
		MC	17	31.18	0.04	0.53	0.26	0.45	0.38	0.10	0.30	B.D.	0.00	0.04	1.50	0.05	1.00	0.01	2.00	B.D.	0.00	0.59	0.20	39.91	0.04
		OC	17	29.74	0.07	0.59	0.32	0.58	0.36	0.24	0.54	0.06	0.00	0.04	1.25	0.09	1.00	0.01	2.00	B.D.	0.00	0.67	0.15	37.20	0.11
		Tot	6	**30.63**	0.08	**0.58**	0.31	**0.50**	0.3	**0.1**	0.5	**0.0**	0.6	**0.0**	1.7	**0.0**	1.0	**0.0**	2.0	**N/**	0.0	**0.6**	0.2	**38.8**	0.1

		al	3						4	**7**	9	**6**	7	**4**	5	**6**	0	**1**	0	**A**	0	**2**	3	**1**	0
91 days	U	HC	11	31.17	0.06	0.48	0.23	0.22	1.36	0.03	0.67	B.D.	0.00	0.03	1.33	0.02	1.50	0.06	0.83	B.D.	0.00	0.77	0.09	38.97	0.11
		IC	10	30.65	0.09	0.46	0.17	0.25	1.16	0.05	0.60	B.D.	0.00	0.03	1.33	B.D.	0.00	0.07	0.57	B.D.	0.00	0.57	0.21	39.56	0.10
		MC	15	30.49	0.11	0.54	0.17	0.16	1.38	0.04	0.50	B.D.	0.00	0.03	1.00	0.03	1.67	0.06	0.50	B.D.	0.00	0.76	0.08	39.12	0.14
		OC	11	27.93	0.12	0.41	0.20	0.27	1.26	0.07	0.71	0.03	1.67	0.07	1.00	0.07	1.29	0.06	0.83	B.D.	0.00	0.57	0.37	34.60	0.11
		Total	47	**30.06**	0.09	**0.47**	0.19	**0.22**	1.27	**0.05**	0.60	**0.03**	0.67	**0.04**	1.00	**0.04**	1.25	**0.06**	0.67	**N/A**	0.00	**0.67**	0.18	**38.06**	0.12
	B	HC	16	29.89	0.20	0.57	0.61	0.44	0.64	0.06	0.33	0.02	1.50	0.12	1.17	0.07	1.14	0.07	0.71	B.D.	0.00	0.46	0.41	38.10	0.20
		IC	17	31.89	0.07	0.58	0.48	0.49	0.51	0.06	0.33	B.D.	0.00	0.05	0.80	0.06	1.00	0.08	0.63	B.D.	0.00	0.45	0.40	40.53	0.09
		MC	17	34.22	0.13	0.48	0.23	0.34	0.85	0.04	0.50	B.D.	0.00	0.03	0.67	0.04	1.25	0.08	0.75	B.D.	0.00	0.42	0.60	44.29	0.15
		OC	18	30.51	0.11	0.32	0.38	0.56	0.54	0.12	1.00	0.01	2.00	0.06	0.83	0.04	2.00	0.08	0.75	B.D.	0.00	0.47	0.40	39.37	0.12
		Total	68	**31.63**	0.13	**0.49**	0.45	**0.46**	0.61	**0.07**	0.71	**0.01**	2.00	**0.06**	1.00	**0.05**	1.40	**0.08**	0.75	**N/A**	0.00	**0.45**	0.44	**40.57**	0.14

180 days	U	HC	8	27.44	0.33	0.42	0.36	0.22	2.14	0.04	0.50	B.D.	0.00	0.08	1.13	0.02	1.50	0.02	1.50	B.D.	0.00	0.70	0.34	34.39	0.30
		IC	6	30.77	0.12	0.49	0.10	0.05	0.60	0.04	0.50	B.D.	0.00	0.05	0.60	0.06	1.00	B.D.	0.00	B.D.	0.00	0.67	0.16	39.14	0.13
		MC	14	29.98	0.09	0.53	0.11	0.06	0.50	0.03	0.33	B.D.	0.00	0.04	0.75	0.03	1.00	B.D.	0.00	B.D.	0.00	0.81	0.11	39.87	0.10
		OC	10	28.94	0.16	0.41	0.29	0.05	0.40	0.05	0.60	0.03	2.00	0.12	1.42	0.14	1.71	0.03	0.67	0.01	1.00	0.73	0.30	37.79	0.15
		Total	38	**29.28**	0.17	**0.46**	0.20	**0.10**	1.40	**0.04**	0.50	**0.03**	0.67	**0.07**	1.14	**0.06**	1.50	**0.03**	0.67	**0.01**	1.00	**0.73**	0.22	**37.80**	0.16
	B	HC	8	31.09	0.13	0.38	0.32	0.33	1.03	0.04	0.25	0.01	3.00	0.06	1.17	0.08	0.88	0.02	1.00	B.D.	0.00	0.67	0.24	39.43	0.16
		IC	11	28.86	0.12	0.38	0.26	0.73	0.59	0.05	0.40	0.03	1.00	0.04	0.75	0.09	1.00	0.02	1.50	B.D.	0.00	0.57	0.33	37.42	0.11
		MC	12	31.22	0.06	0.48	0.13	0.49	0.53	0.05	0.20	B.D.	0.00	0.03	1.33	0.02	2.00	0.01	3.00	B.D.	0.00	0.58	0.31	41.40	0.04
		OC	10	32.61	0.04	0.47	0.38	0.64	0.31	0.11	0.82	B.D.	0.00	0.07	0.57	0.07	1.00	0.01	2.00	B.D.	0.00	0.57	0.46	41.07	0.05
		Total	41	**30.94**	0.09	**0.43**	0.26	**0.54**	0.57	**0.06**	0.67	**0.02**	1.00	**0.05**	1.00	**0.07**	1.00	**0.02**	1.00	**N/A**	0.00	**0.60**	0.33	**39.83**	0.09
36	U	HC	3	29.45	0.05	0.53	0.21	0.03	0.3	0.0	0.6	0.0	0.0	0.0	0.4	0.0	1.5	0.0	1.0	0.0	0.5	0.7	0.0	37.2	0.0

5									3	3	7	0	0	7	3	2	0	3	0	2	0	6	1	2	5
days		IC	7	27.89	0.08	0.48	0.13	0.06	0.17	0.02	0.50	B. D.	0.00	0.04	0.50	0.00	0.00	0.02	2.00	B. D.	0.00	0.69	0.16	34.53	0.09
		MC	4	30.25	0.05	0.49	0.10	0.04	0.25	0.02	0.50	B. D.	0.00	0.03	0.67	0.00	0.00	0.01	3.00	B. D.	0.00	0.75	0.17	38.12	0.05
		OC	2	29.41	0.08	0.43	0.12	0.04	0.00	0.01	0.00	0.02	1.00	0.07	0.14	0.00	0.00	0.03	1.33	0.01	1.00	0.70	0.43	38.43	0.03
		Total	16	**29.25**	0.06	**0.48**	0.15	**0.04**	0.25	**0.02**	0.50	**0.01**	1.00	**0.05**	0.40	**0.00**	0.00	**0.02**	1.50	**0.01**	1.00	**0.73**	0.18	**37.07**	0.05
	B	HC	11	30.65	0.08	0.30	0.27	0.76	0.22	0.02	0.50	B. D.	0.00	0.05	1.00	0.03	1.00	0.02	1.50	B. D.	0.00	0.76	0.18	38.30	0.07
		IC	11	30.64	0.05	0.35	0.17	0.80	0.09	0.03	0.67	B. D.	0.00	0.05	0.80	0.02	1.50	0.02	1.50	B. D.	0.00	0.51	0.18	37.87	0.05
		MC	12	32.08	0.03	0.45	0.20	0.64	0.17	0.02	1.00	B. D.	0.00	0.04	0.75	0.02	1.50	0.04	0.75	B. D.	0.00	0.70	0.16	38.03	0.32
		OC	14	31.81	0.07	0.35	0.23	0.73	0.12	0.09	0.78	B. D.	0.00	0.03	0.67	0.03	1.00	0.02	1.50	B. D.	0.00	0.77	0.22	40.41	0.07
		Total	48	**31.30**	0.06	**0.36**	0.22	**0.73**	0.15	**0.04**	0.75	**N/A**	0.00	**0.04**	1.00	**0.02**	1.50	**0.03**	1.00	**N/A**	0.00	**0.68**	0.19	**38.65**	0.12
Neol.	B	HC	10	35.92	0.19	0.52	0.19	0.34	1.03	0.02	2.00	0.10	0.90	0.26	1.12	0.35	1.37	0.00	1.38	0.01	1.00	0.01	2.00	48.11	0.18

		IC	12	38.98	0.08	0.48	0.15	0.26	0.77	0.03	1.00	0.06	0.00	0.18	0.78	0.24	1.50	0.01	1.49	B.D.	0.00	0.03	1.00	50.54	0.07
		MC	12	39.13	0.12	0.57	0.23	0.17	1.59	B.D.	N/A	0.04	2.00	0.10	0.70	0.04	1.50	0.01	1.30	B.D.	0.00	B.D.	0.00	52.13	0.11
		OC	13	36.15	0.29	0.33	0.48	0.30	0.83	0.19	2.84	0.74	0.00	1.39	2.97	3.09	3.08	0.01	3.08	0.04	1.00	0.15	3.00	48.22	0.29
		Total	47	**37.54**	0.17	**0.47**	0.23	**0.27**	1.00	**0.08**	1.88	**0.24**	0.00	**0.48**	2.42	**0.93**	2.80	0.01	1.81	**0.02**	1.00	**0.06**	2.17	**49.75**	0.16
